# Chimpanzee histology and functional brain imaging show that the paracingulate sulcus is not human-specific

**DOI:** 10.1038/s42003-020-01571-3

**Published:** 2021-01-08

**Authors:** Céline Amiez, Jérôme Sallet, Jennifer Novek, Fadila Hadj-Bouziane, Camille Giacometti, Jesper Andersson, William D. Hopkins, Michael Petrides

**Affiliations:** 1 Univ Lyon, Université Lyon 1, Inserm, Stem Cell and Brain Research Institute U1208, 69500 Bron, France; 2 Wellcome Integrative Neuroimaging Centre, Department of Experimental Psychology, University of Oxford, Oxford, OX1 3SR UK; 3 Montreal Neurological Institute, Department of Neurology and Neurosurgery, McGill University, Montreal, Quebec, Canada; 4 Integrative Multisensory Perception Action & Cognition Team (ImpAct), INSERM U1028, CNRS UMR5292, Lyon Neuroscience Research Center (CRNL), Lyon, France, University of Lyon 1, Lyon, France; 5 Wellcome Integrative Neuroimaging Centre, fMRIB, University of Oxford, Headington, UK; 6 Department of Comparative Medicine, University of Texas MD Anderson Cancer Center, Bastrop, TX 78602 USA; 7 Montreal Neurological Institute, Department of Neurology and Neurosurgery and Department of Psychology, McGill University, Montreal, Quebec, Canada

**Keywords:** Cognitive neuroscience, Evolution

## Abstract

The paracingulate sulcus -PCGS- has been considered for a long time to be specific to the human brain. Its presence/absence has been discussed in relation to interindividual variability of personality traits and cognitive abilities. Recently, a putative PCGS has been observed in chimpanzee brains. To demonstrate that this newly discovered sulcus is the homologue of the PCGS in the human brain, we analyzed cytoarchitectonic and resting-state functional magnetic resonance imaging data in chimpanzee brains which did or did not display a PCGS. The results show that the organization of the mid-cingulate cortex of the chimpanzee brain is comparable to that of the human brain, both cytoarchitectonically and in terms of functional connectivity with the lateral frontal cortex. These results demonstrate that the PCGS is not human-specific but is a shared feature of the primate brain since at least the last common ancestor to humans and great apes ~6 mya.

## Abstract (web summary)

The paracingulate sulcus (PCGS) is a brain structure long thought to be specific to humans, and variation in this structure has been linked to personality traits and cognitive abilities. In this study, Céline Amiez and Jérôme Sallet et al. analyze brain imaging data from humans and chimpanzees to demonstrate that the PCGS is in fact present in our closest relative and its functional connectivity in chimpanzees is comparable to that in humans.

## Introduction

**Fig. 1:**
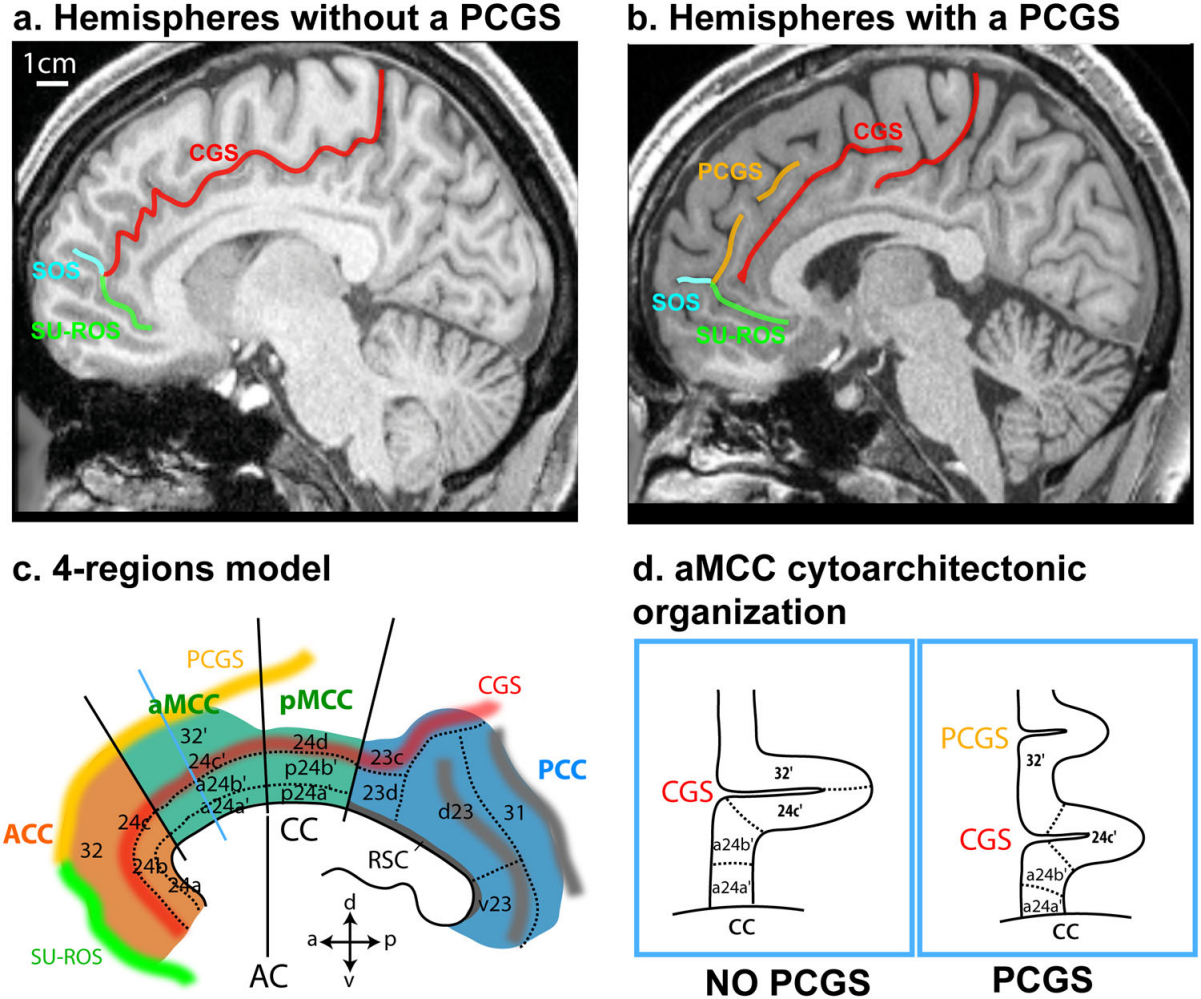
Morphological and cytoarchitectonic organization of the cingulate cortex in hemispheres without or with a PCGS in the human brain. **a** In hemispheres displaying no PCGS, the CGS starts at the intersection with the supra-rostral sulcus (SUROS) and the sulcus sus-orbitalis (SOS) in front of the genu of the corpus callosum. **b** In hemispheres with a PCGS, it is the PCGS that starts rostrally at the intersection with the SUROS and the SOS^13,14^. **c** The 4-regions model is represented in a hemisphere displaying a PCGS. This model identifies the limit between the ACC and the aMCC at the level of the anterior limit of the genu of the corpus callosum, the limit between the aMCC and the pMCC as being the anterior commissure. In the aMCC, when a PCGS is present, both banks of the CGS are occupied by area 24c′ whereas the ventral bank of the PCGS is occupied by area 32′. When a PCGS is absent, the ventral and dorsal banks of the CGS are respectively occupied by area 24c′ and 32′. **d** Cytoarchitectonic organization of the aMCC in hemispheres with and without a PCGS, as shown on coronal sections at the anteroposterior level displayed by the blue line in (**c**). a anterior, p posterior, d dorsal, v ventral, AC anterior commissure, cc corpus callosum, ACC anterior cingulate cortex, CGS cingulate sulcus, MCC mid-cingulate cortex, PCC posterior cingulate cortex, RSC retrosplenial cortex, PCGS paracingulate sulcus, SU-ROS supra-rostral sulcus, SOS sulcus sus-orbitalis. Figure 1c modified from Supplementary Fig. 3 in Amiez et al.^13^.

Understanding the mechanisms underlying brain evolution, and more specifically of the human brain, is still the topic of intense debates^1–4^. Comparative neuroanatomical studies have demonstrated that ecological and social pressures are key factors that have driven the expansion of the neocortex in primates. But this expansion has differentially impacted brain circuits^5^. With the development of neuroimaging tools, one could address comparative neuroanatomy questions in vivo at different levels of analysis, from gross morphology (e.g., sulcal pattern analysis) to brain connectivity (e.g., resting-state functional Magnetic Resonance Imaging analysis). Comparative neuroimaging studies are principally relying on a comparison between human brains and a limited number of non-human primate models, namely macaques and marmosets^6,7^, whose ancestors diverged from human ancestors 25 and 35 million years ago, respectively^8^. With a common evolutionary history until 7 million years ago, the chimpanzee is a key model for better understanding the evolution of brain regions that have largely expanded in the human brain, such as the medial prefrontal cortex^9^. Among the sulci that characterize the human medial frontal cortex, the paracingulate sulcus (PCGS) is a secondary sulcus running dorsal and parallel to the cingulate sulcus (CGS) in a rostro-caudal direction^10,11^ in the medial frontal cortex. The PCGS is observed in about 70% of subjects at least in one hemisphere^10–13^ and most often starts at the intersection with the sus-orbitalis and the supra-rostral sulcus, in front and at the level of the anterior limit of the genu of the corpus callosum, where the anterior cingulate cortex (ACC) lies (Fig. 1a, b)^13,14^. We are referring here to the cingulate subdivisions proposed by Vogt et al.^15^ (Fig. 1c). From the ACC, the PCGS runs caudally where the anterior mid-cingulate cortex (aMCC) lies, but it can also run as far posterior as the level of the anterior commissure (where the posterior mid-cingulate cortex (pMCC) lies) (Fig. 1). In the human brain, the impact of the presence of a PCGS on the cytoarchitectonic organization (i.e., the cellular organization of the cerebral cortex) of the aMCC is known^10^: when the PCGS is absent, areas 24c′ and 32′ occupy, respectively, the ventral and the dorsal banks of the CGS; however, when a PCGS is present, area 24c′ occupies both banks of the CGS and area 32′ occupies the PCGS (Fig. 1).

In the human brain, the morphological variability of the PCGS has been linked to personality traits^16–18^, and pathologies^19–23^, and has also been associated with higher-order cognitive processing, i.e., several so-called human-specific processes^12,24–27^. These observations have led some investigators to suggest that cortical area 32′ which occupies the PCGS when present and the dorsal bank of the CGS when the PCGS is absent, might be unique to the human brain^28,29^. However, a recent study has shown, based on morphological observations of the sulcal organization on structural MRI scans, the presence of a putative homologue of the human PCGS in 33.8% of chimpanzees, at least in one hemisphere^13^. Furthermore, opposing the view of a lack of area 32′ in non-human primates, this transition area (i.e., area 32′) between the cingulate cortex and cortex of the medial frontal gyrus has been shown in macaques^30^. However, the statement that the PCGS is not human-specific and can be observed in chimpanzee brains must be supported by cytoarchitectonic evidence showing that the organization of this region in the chimpanzee brain is comparable to that in the human brain.

In the chimpanzee brain, it is not known whether, as in the human brain, the PCGS starts in the ACC and runs caudally to the midcingulate cortex (MCC)^13^. In the present study, we therefore first assessed the extent of the PCGS in both human and chimpanzee brains and hypothesized that, if the sulcus that we identified as a PCGS in the chimpanzee^13^ is homologous to the human PCGS, the mapping of the cytoarchitectonic organization of the aMCC in the chimpanzee should be comparable to that in the human brain.

It should be noted that, in the human brain, the functional connectivity within the MCC of seeds located in the PCGS and in the CGS, when the PCGS is absent, is similar. Specifically, Loh et al.^31^ have assessed the functional connectivity of the anterior rostral cingulate zone (RCZa) which is located within the anterior part of the MCC. Within the RCZa, there are limb and face motor representations with the limb motor representations lying within the CGS even when a PCGS is present; the face motor representations lie in the PCGS if present and in the CGS if the PCGS is absent^32^. Loh et al.^31^ have shown that the functional connectivity of the face motor representation of RCZa with lateral prefrontal and lateral motor regions of interest is similarly organized, regardless of whether it is located in the CGS in hemispheres without a PCGS or in the PCGS in hemispheres with a PCGS. The functional connectivity is stronger with anterior prefrontal regions and weaker with posterior motor regions^31^.

In the present study, we examined (1) the extent of the PCGS in both chimpanzee and human brains, (2) the cytoarchitectonic organization of the aMCC with a specific emphasis on the distribution of areas 24c′ and 32′ in three post-mortem chimpanzee brains which did or did not display a PCGS, and (3) in vivo functional connectivity of this region in four anesthetized chimpanzees based on the availability of resting-state functional magnetic resonance imaging (rs-fMRI) data. The results demonstrate that, in the chimpanzee brain, the impact the PCGS has on the cytoarchitectonic organization of the aMCC is comparable to that observed in the human brain. The results also show that the functional connectivity of the CGS and the PCGS is comparable to that observed in the human brain^31^. Altogether, these results demonstrate that the PCGS in chimpanzee brains is comparable in terms of cytoarchitecture and functional connectivity with the PCGS in human brains. These observations demonstrate that the PCGS is not human-specific and had already emerged in the brains of the last common ancestor with chimpanzees.

## Results

### Morphological study

**Fig. 2:**
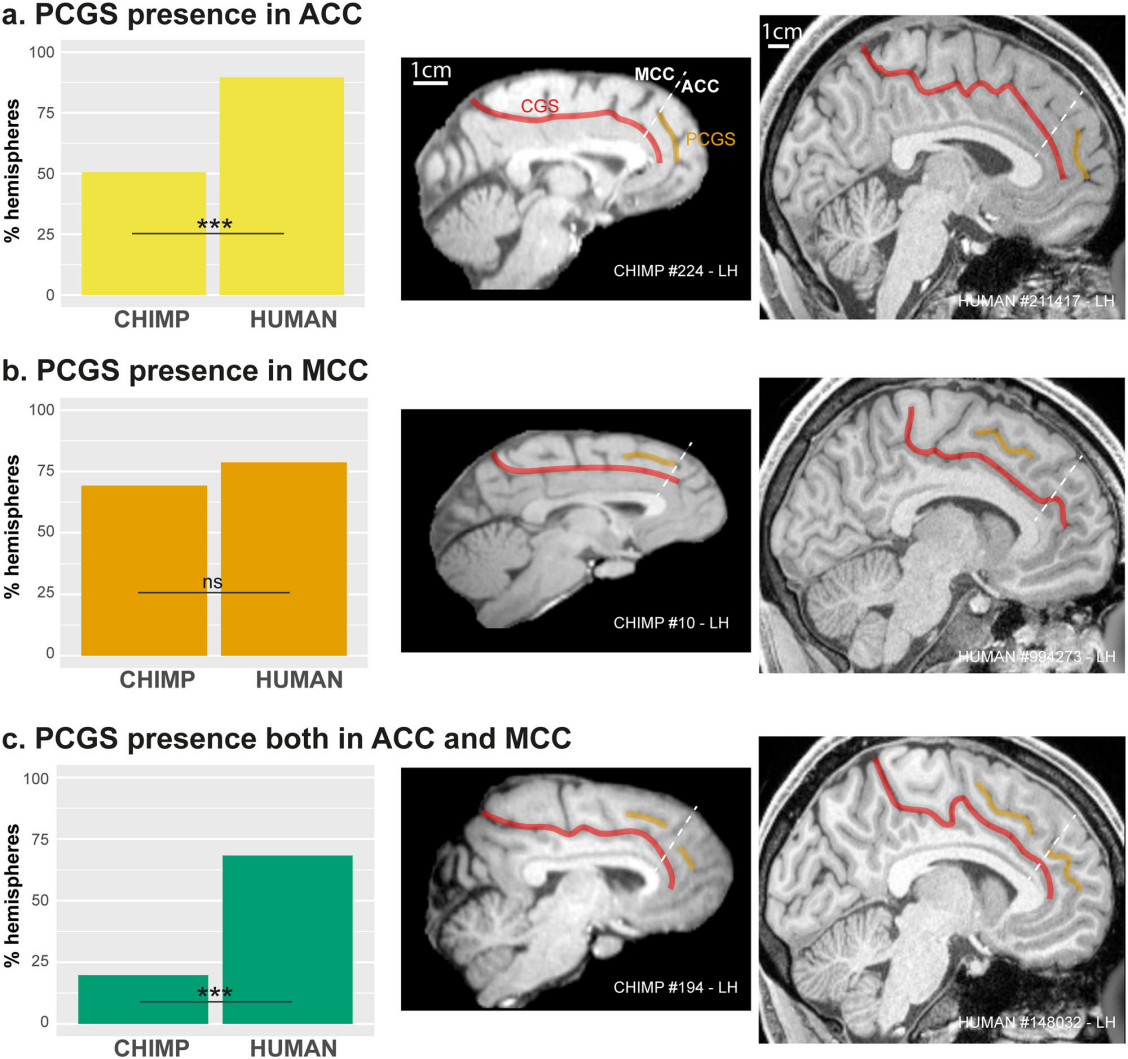
Occurence of the PCGS in the ACC and MCC in the chimpanzee and the human brains. Probability of occurrence of a PCGS in the ACC (**a**), the MCC (**b**), or in both ACC and MCC (**c**) in chimpanzee versus human brains. The putative limit between ACC and MCC is represented by the dashed line. CGS and PCGS correspond to the red and yellow lines, respectively. Left diagrams show that, in hemispheres displaying a PCGS (i.e., in *n* = 183 human brain hemispheres and *n* = 91 chimpanzee brain hemispheres), the probability of occurrence of a PCGS in the ACC as well as in both the ACC and the MCC is higher in human than in chimpanzee brains (dependent variable: PCGS present (0/1), main effect species: *χ*^2 ^= 49.7, df = 1, *p*-value = 1.79e−12, logistic regression). By contrast, the probability of occurrence of a PCGS in the MCC is similar in human and chimpanzee (dependent variable: PCGS present in MCC (0/1), main effect species: *χ*^2 ^= 2.9, df = 1, *p*-value = 0.09, logistic regression). ACC anterior cingulate cortex, LH left hemisphere, MCC mid-cingulate cortex, PCGS paracingulate sulcus, ***p < 0.001, logistic regression; ns non-significant logistic regression.

We first re-analyzed data from Amiez et al.^13^ to assess the occurrence of a PCGS in the ACC versus the MCC in 197 human and 225 chimpanzee brains. Note that the ACC/MCC limit was identified using the probabilistic cytoarchitectonic map of the ACC from the JuBrain atlas (see “Methods”)^33^. This new analysis demonstrates that, in hemispheres displaying a PCGS (i.e., *n* = 183 human brain hemispheres, *n* = 91 chimpanzee brain hemispheres), the probability of observing a PCGS in the ACC is higher (89.6%) in human hemispheres than in chimpanzee (50.5%) hemispheres (Fig. 2a, dependent variable: PCGS present (0/1), main effect species: *χ*^2^ = 49.7, df = 1,*p*-value = 1.79e−12, logistic regression; source data are provided as Supplementary data 1).

By contrast, the probability of observing a PCGS in the MCC is comparable in human (78.7%) and chimpanzee (69.2%), i.e., it does not statistically differ between these two species (Fig. 2b) (dependent variable: PCGS present in MCC (0/1), main effect species: *χ*^2^ = 2.9, df = 1, *p*-value = 0.09, logistic regression). Finally, the probability of observing a PCGS in both the ACC and the MCC is significantly higher in human (in 68.3% of hemispheres displaying a PCGS) compared to chimpanzees (in 19.8% of hemispheres displaying a PCGS, Fig. 2c) (dependent variable: PCGS location (ACC/MCC), main effect species: *χ*^2^ = 60.2, df = 1, *p*-value = 8.48e−15, logistic regression).

### Cytoarchitectonic study

Note that our analysis is specifically focused on the distribution of area 24c′ and area 32′ and the impact of the presence/absence of a PCGS on it. Previous studies had already investigated the dorsal-ventral or rostro-caudal organization of the cingulate cortex and adjacent areas of the medial frontal region^7,33–36^.

From the morphological inspection of the three chimpanzee brains included in the following cytoarchitectonic analysis, we selected four hemispheres. We analyzed the left hemisphere of CHIMP_1 and the right hemisphere of CHIMP_3 in which the PCGS was absent. We also selected the right hemisphere of CHIMP_1 and the left hemisphere of CHIMP_2 which displayed a PCGS. In CHIMP_2, the PCGS was present in the anteriormost part of the aMCC, but absent in the posterior part of the aMCC. Note that the remaining hemispheres (left hemisphere of CHIMP_3 and right hemisphere of CHIMP_2) displayed no PCGS.

#### Hemispheres without a PCGS

**Fig. 3:**
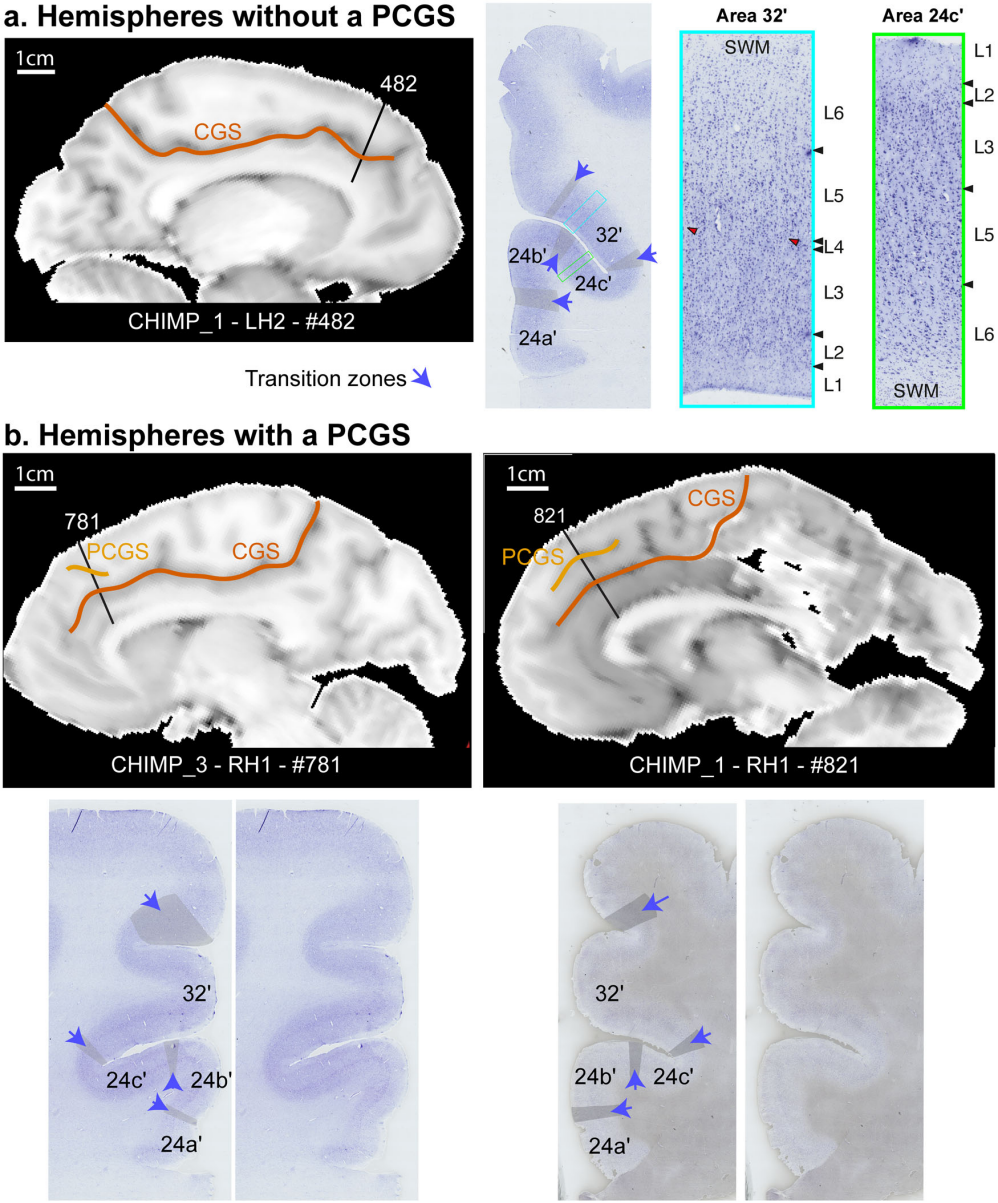
Impact of the presentce of a PCGS on the cytoarchitectonic organization of the anterior MCC. Cytoarchitectonic organization of the anterior MCC in hemispheres without (**a**) and with (**b**) a PCGS. **a** The MCC of the left hemisphere of CHIMP_1 is presented on a sagittal view of a post-mortem MRI scan (left panel). The CGS is marked in red. The coronal section presented on the middle panel corresponds to the antero-posterior level defined by a black line on the MRI image (slice 482). The right panels present the labeled and raw Nissl-stained slices. The black lines represent the limits between areas. The gray zones identified by a blue arrow correspond to transition zones between two adjacent cytoarchitectonic areas. Area 24c′ occupies the ventral bank of the CGS and area 32′ occupies the dorsal bank of the CGS. The photomicrographs of area 32′ (corresponding to the region identified by a blue box on the coronal section) and area 24c′ (corresponding to the region identified by a green box on the coronal section) are displayed on the right panels. Results show the presence of a dysgranular layer 4 (in red are displayed the granular patches) in area 32′ and the absence of this layer in area 24c′. **b** The MCC of the right hemispheres of CHIMP_3 and CHIMP_1 are presented on sagittal views of post-mortem MRI scans. The CGS is marked in red, the PCGS in orange. The coronal sections presented on each Nissl-stained slices correspond to the antero-posterior levels defined by a black line on the MRI images (CHIMP_3: slice 781, CHIMP_1: slice 821). In both chimpanzees, area 24c′ occupies the ventral bank, the fundus, and the lateral-most part of the dorsal bank of the CGS. Area 32′ occupies the dorsal bank of the CGS, the gyrus between the CGS and the PCGS and the ventral bank of the PCGS. CGS cingulate sulcus, MRI magnetic resonance imaging, PCGS paracingulate sulcus, L1-6 cytoarchitectonic layers 1-6, SWM superficial white matter.

**Fig. 4:**
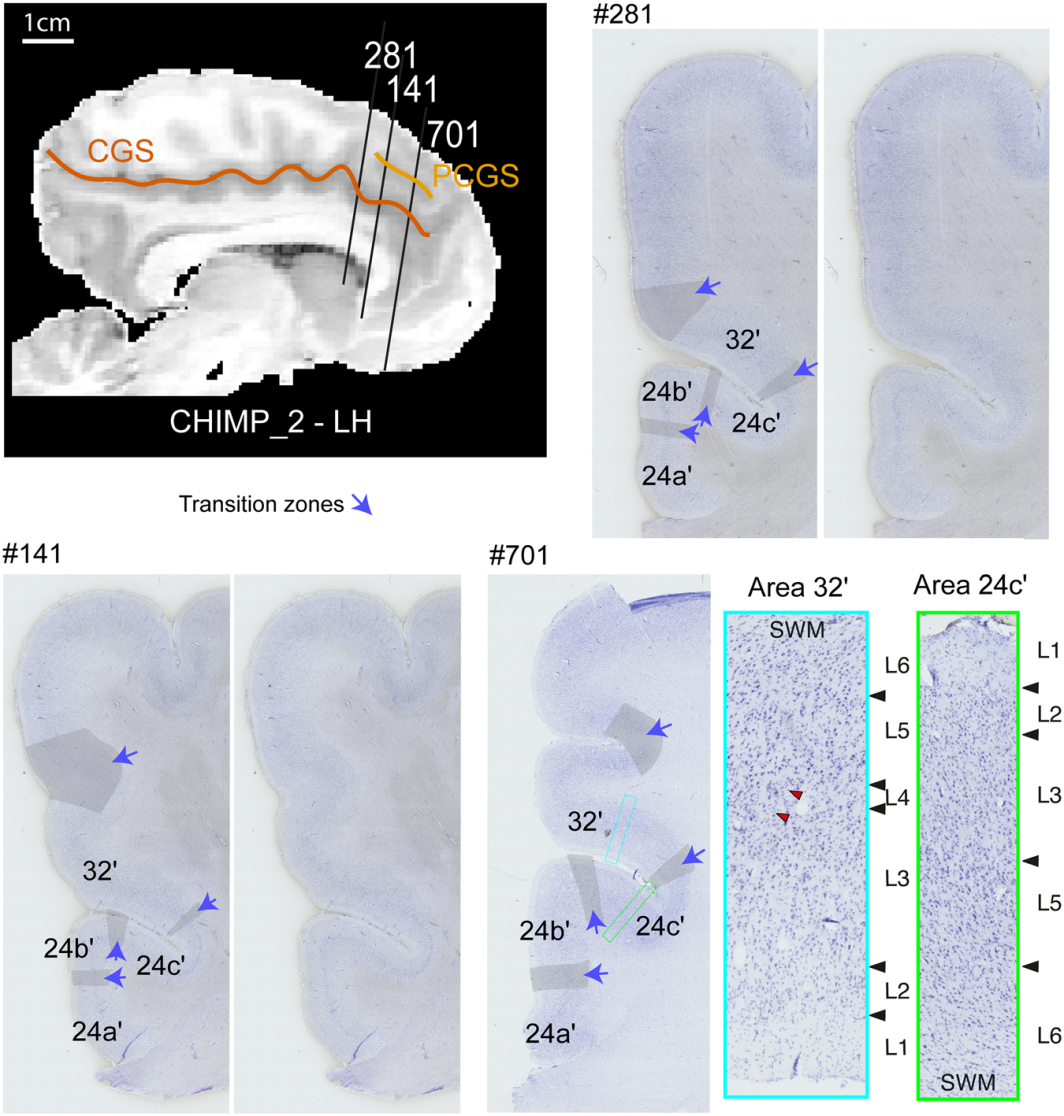
Cytoarchitectonic organization of the anterior MCC of a hemisphere displaying a PCGS in its anterior part and no PCGS in its posterior part. The MCC of the left hemisphere of CHIMP_2 is presented on a sagittal view of a post-mortem MRI scan. The CGS is marked in red, the PCGS in orange. The Nissl-stained coronal sections presented correspond to the antero-posterior levels defined by a black line on the MRI images (slice 281 where the PCGS is absent, slices 141 and 701 where the PCG is present). On slice 281 where the PCGS is absent, (1) area 24c′ occupies the ventral bank, the fundus, and the lateral-most part of the dorsal bank of the CGS, (2) area 32′ occupies the dorsal bank of the CGS, the gyrus between the CGS and the PCGS and the ventral bank of the PCGS. On slices 141 and 701 where the PCGS is present, (1) area 24c′ occupies the ventral bank, the fundus, and the lateral-most part of the dorsal bank of the CGS, (2) area 32′ occupies the dorsal bank of the CGS, the gyrus between the CGS and the PCGS and the ventral bank of the PCGS. The gray zones identified by a blue arrow correspond to transition zones between two adjacent cytoarchitectonic areas. The photomicrographs of area 32′ (corresponding to the region identified by a blue box on the coronal section of slice #701) and area 24c′ (corresponding to the region identified by a green box on the coronal section of slice #701) are displayed on the right panels. Results show the presence of a dysgranular layer 4 (in red are displayed the granular patches) in area 32′ and the absence of this layer in area 24c′. CGS cingulate sulcus, PCGS paracingulate sulcus, LH left hemisphere, L1-6 cytoarchitectonic layers 1-6, SWM superficial white matter.

We examined first the MCC within the left hemisphere of CHIMP_1, which did not display a PCGS. As shown in Fig. 3a, proceeding from the corpus callosum dorsally, we observed successively areas 24a′ and 24b′, respectively on the ventral and dorsal part of the gyrus of the cingulate cortex, and areas 24c′ and 32′, respectively in the ventral and dorsal bank of the CGS (for the cytoarchitectonic characteristics, see “Methods” section). Note that a transition zone was observed between each area (i.e. between areas 24a′ and 24b′, between areas 24b′ and 24c′, and between areas 24c′ and 32′). Proceeding dorsally along the cingulate gyrus, the cytoarchitecture does not change abruptly, but rather a smooth reorganization is observed. We also examined the posterior part of the aMCC of CHIMP_2 (Fig. 4, slice 81) which does not display a PCGS (although the anterior part of the aMCC does possess a PCGS). The results demonstrated exactly the same cytoarchitectonic organization as in the left MCC of CHIMP_1.

#### Hemispheres with a PCGS

We examined the right hemisphere of CHIMP_1 and CHIMP_3, both of which display a PCGS in the MCC. In both chimpanzees, we observed successively from the corpus callosum in a dorsal direction towards the lateral cortical surface, area 24a′ and area 24b′, respectively on the ventral and dorsal parts of the cingulate gyrus, area 24c′ in the ventral bank and in part of the dorsal bank of the CGS, and area 32′ which extends from a part of the dorsal bank of the CGS to the ventral bank of the PCGS (for the cytoarchitectonic characteristics, see the “Methods” section). As in hemispheres in which the PCGS is absent, we observed small transition zones between adjacent areas (Fig. 3b). We also examined the anterior part of the aMCC in the left hemisphere of CHIMP_2 (Fig. 4, slices 141 and 701) which displays a PCGS. The results demonstrated exactly the same cytoarchitectonic organization as in the right MCC of CHIMP_1 and CHIMP_3.

### Rs-fMRI study

From the morphological inspection of the four chimpanzee brains included in the following rs-fMRI analysis, we observed a PCGS in the left hemisphere of CHIMP_A, and in both the left and right hemispheres of CHIMP_C. The right hemisphere of CHIMP_A, and both hemispheres of CHIMP_B and CHIMP_D did not display a PCGS.

**Fig. 5:**
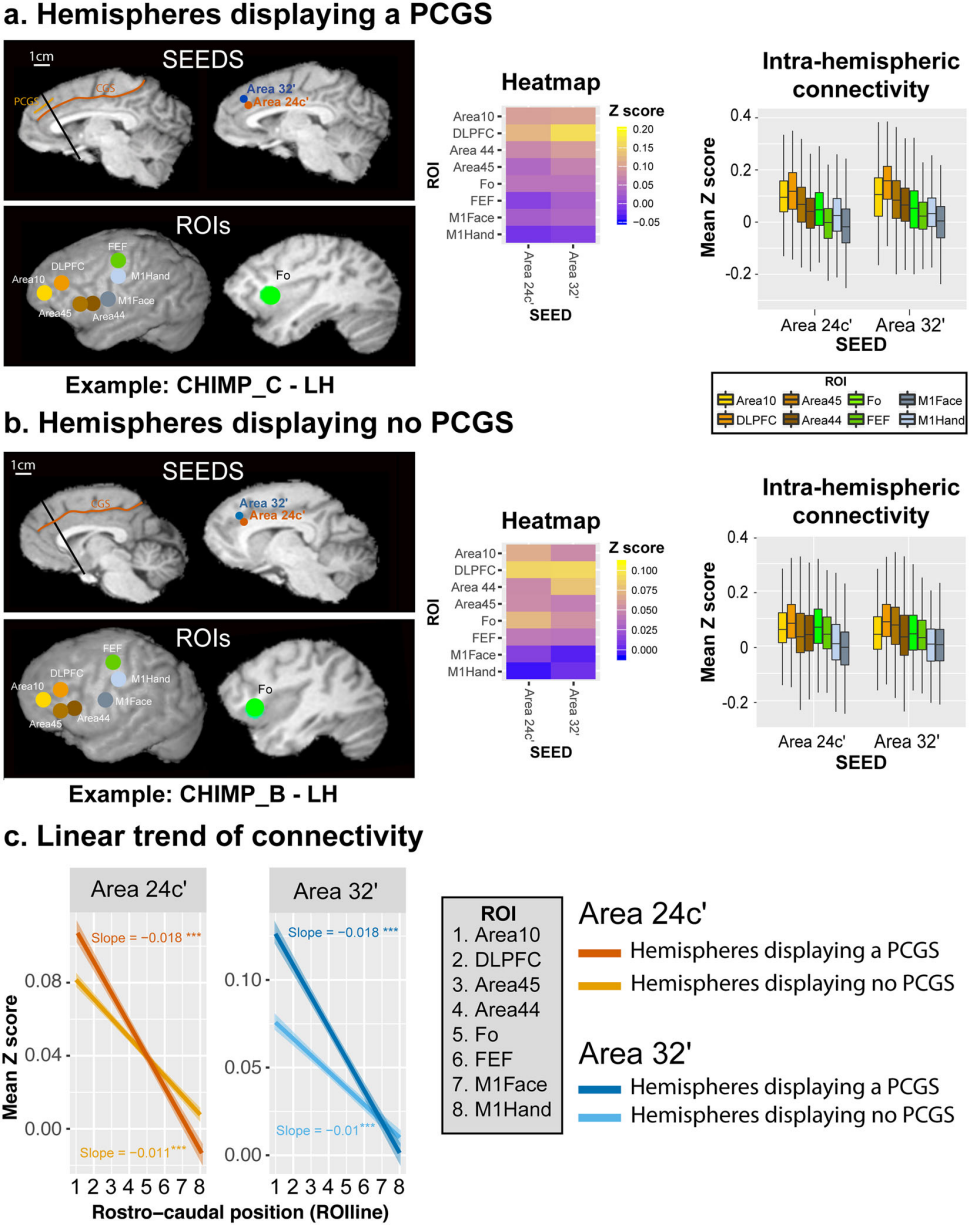
Intra-hemispheric rostro-caudal functional organization between areas 24c′ and 32′ with the lateral frontal cortex in hemispheres displaying a PCGS (a, *N* = 3) and no PCGS (b, *N* = 5). **a** The location of each seed is shown in a typical example of a hemisphere displaying a PCGS (CHIMP_C – LH). The location of each region of interest (ROI) is shown on the cortical surface of the same hemisphere. The heat-map represents the averaged seed-ROI *Z* values in hemispheres displaying a PCGS. Boxplots displaying the mean ± SD Z-transformed connectivity between each seed (areas 24c′ and 32′) with the various ROIs in hemispheres displaying a PCGS. Results show that the correlation strength between Area 24c′ and Area 32′ with the prefrontal cortex is significantly higher than with the motor zones (Area 24c′: df = 7, *F* = 154.8, *p* < 2.2e−16; Area 32′: df = 7, *F* = 157, *p* < 2.2e−16, ANOVA). **b** The location of each seed is shown in a typical example of a hemisphere displaying no PCGS (CHIMP_B – LH). The heat-map represents the averaged seed-ROI *Z* values in hemispheres displaying a PCGS. Boxplots displaying the mean ± SD Z-transformed connectivity between each seed (areas 24c′ and 32′) with the various ROIs in hemispheres displaying no PCGS. Results show that the correlation strength between Area 24c’ and Area 32′ with the prefrontal areas is significantly higher than with the motor zones (Area 24c′: df = 7, *F* = 123.5, *p* < 2.2e−16; Area 32’: df = 7, *F* = 110.1, *p* < 2.2e−16, ANOVA). **c** Significant negative linear trend of connectivity (slope) of each seed with the rostral-caudal position of lateral frontal ROIs (ROIlines) in hemispheres displaying or not a PCGS. The ROIline was obtained by recoding the ROIs in terms of their relative rostro-caudal rank: 1, Area 10; 2, DLPFC; 3, Area 45; 4, Area 44; 5, Fo; 6, FEF; 7, M1Face; 8, M1Hand. Results show that these negative slopes were statistically similar for both seeds (Area 24c′ and Area 32′) and for both morphologies (presence or absence of a PCGS) (interaction between seed identity, seed location, and ROIline, *F* = 3.03, *p* > 0.05, ns, 3-ways ANOVA). LH left hemisphere; *** statistically significant at *p* < 0.001.

We first assessed, in hemispheres displaying a PCGS, the intra-hemispheric functional connectivity profiles of areas 24c′ and 32′ with Regions Of Interest (ROIs) located in the lateral frontal cortex (Fig. 5a). To be able to conduct a comparison of connectivity fingerprints between species, ROIs were chosen for their known homologies between chimpanzee and human brains. The location of seeds and ROIs are displayed on the medial (left diagram) and the lateral cortical surface (right diagram) of a typical example (left hemisphere of CHIMP_C). The heat-maps reflecting the average correlation strength between each pair of seed-ROI clusters in the three hemispheres displaying a PCGS (see “Methods”) are shown in Fig. 5a. The Boxplots further depict the average *Z* values of correlations between the two seeds and ROIs across the three hemispheres displaying a PCGS (see “Methods”). The results demonstrate how the activity of each seed is differentially correlated with the activity of lateral prefrontal/motor ROIs (see “Methods”). For each seed, we tested these differences in connectivity *z* values with a generalized linear model with ROI zones (prefrontal zones: Area 10, DLPFC (dorsolateral prefrontal cortex), Area 45, Area 44, and Fo (Frontal operculum), and motor zones: FEF (Frontal Eye Field), M1Face and M1Hand) as a fixed effect. To account for the variability observed across individuals, the chimpanzee ID was used as a random effect. The results indicated that, as in the human brain, the correlation strength between Area 24c′ and Area 32′ with the prefrontal cortex is significantly higher than with the motor zones (Area 24c′: df = 7, *F* = 154.8, *p* < 2.2e−16; Area 32’: df = 7, *F* = 157, *p* < 2.2e−16, ANOVA). We then assessed the linearity of correlation trends with lateral frontal areas along the rostro-caudal axis (Fig. 5c). To test and quantify these linear trends from anterior prefrontal to motor areas, we recoded the various lateral frontal ROIs into a numeric axis variable (ROIline) that corresponded to their relative posterior-to-anterior positions (see “Methods”). Based on this coding, the lowest value (1) corresponds to Area 10 (the most anterior ROI) and the highest value (8) corresponds to the M1-Hand region (the most posterior ROI).

We performed the same analysis in hemispheres without a PCGS. The location of seeds and ROIs are displayed on the medial (left diagram) and the lateral cortical surface (right diagram) of a typical example (left hemisphere of CHIMP_B). The results indicated that, as in hemispheres displaying a PCGS, the correlation strength between Area 24c′ and Area 32′ with the prefrontal areas is significantly higher than with the motor zones as demonstrated by heat-maps and boxplots (Fig. 5b, Area 24c′: df = 7, *F* = 123.5, *p* < 2.2e−16; Area 32′: df = 7, *F* = 110.1, *p* < 2.2e−16, ANOVA; source data are provided as Supplementary data 2).

We then performed multiple linear regressions on the correlation values with seed identity, seed location, and ROIline as predictors. A significant negative linear trend (slope) was observed for both seeds (stronger correlation with rostral prefrontal areas) and in both morphology types (presence or absence of a PCGS) (Fig. 5Cc. These negative slopes were statistically similar for both seeds (Area 24c′ and Area 32′) and for both morphologies (presence or absence of a PCGS) (interaction between seed identity, seed location, and ROIline, *F* = 3.03, *p* = 0.08, ns, 3-ways ANOVA).

Thus, the connectivity profiles of areas 24c′ and 32′ with the lateral frontal cortex regions follow the same pattern in hemispheres with a PCGS and those hemispheres that do not display a PCGS: both areas 24c′ and 32′ display equally stronger functional coupling with the lateral prefrontal cortex and weaker with the motor cortex.

## Discussion

The present study demonstrates that the PCGS in the common chimpanzee (*Pan troglodytes*), as previously identified by morphological examination^13^, does correspond to the PCGS observed in the human (*Homo sapiens sapiens*)^10,11^, both cytoarchitectonically and in term of functional connectivity.

When a PCGS is absent, the ventral and dorsal banks of the CGS are, respectively, occupied by areas 24c′ and 32′; when a PCGS is present, we observed an expansion of area 32′ on the medial wall above the CGS up to the fundus of the PCGS. Importantly, the functional connectivity of both areas 24c′ and 32′ with the lateral frontal cortex is similarly organized to that in the human brain^31^: both areas display equally stronger connectivity with rostral prefrontal areas than with caudal motor areas. Importantly, this gradient of functional connectivity can be observed despite the positioning of each ROI on the basis of the sulcal morphological organization in each chimpanzee brain (see “Methods” and Supplementary Fig. 1). Specifically, the positioning of each ROI was based on (1) the fMRI literature on the human brain concerning the precise relationship between the local sulcal organization and functional activity in the regions of interest^37–41^, and (2) studies revealing that the sulcal organization between human and chimpanzee is well preserved^13,37,42,43^. We chose this methodology because, for both ethical and methodological considerations, there is no study assessing the direct relationships between local sulcal morphology and functional activity in behaving chimpanzees. The present study strongly suggests that, in addition to preservation of the sulcal and cytoarchitectonic organization from the chimpanzee to the human brain, the sulcal-functional organization is also preserved. This is of importance because it indicates that the understanding of the sulcal organization in great apes may allow us to infer the functional organization of the brain in chimpanzees.

Based on the morphological sulcal organization of the medial prefrontal cortex^13^ and on the present study, three differences can be identified between the human and the chimpanzee cingulate organization: (1) the PCGS is present in fewer hemispheres in the chimpanzee (33.8% of chimpanzees display a PCGS at least in one hemisphere, compared to about 70% of humans); (2) the PCGS is more frequently observed in the left hemisphere than in the right hemisphere in the human but not in the chimpanzee brains; (3) the PCGS has a more caudal distribution in chimpanzee than in human brains, implying that the PCGS is more commonly found in the MCC compared to the ACC in chimpanzees.

The anatomo-functional organization of the cingulate cortex has received considerable attention. Along the rostral-caudal axis, several anatomical and functional subdivisions have been identified^15,44–50^. Similarly, differences between the cingulate gyrus and the CGS have been demonstrated^44,51–53^. One could identify similar functional properties of neurons in the ventral and dorsal banks of the CGS. For instance, neurons sensitive to distance to reward in a reward-guided sequential task have been recorded in both banks of the CGS^54,55^. However, very few studies have directly investigated what might be the respective roles of areas 24c′ and 32′ that occupy the banks of the CGS. In a rare study investigating the functional properties of the dorsal and ventral banks of the CGS in macaques, major differences were reported^56^. Only neurons in the dorsal bank were modulated by the oculomotor saccade direction and, in addition, neurons in the dorsal bank were most active prior to the choice, while neurons in the ventral bank were most active at the outcome phase.

Sensorimotor properties of the human cingulate cortex have been well characterized^57^. The aMCC contains a cingulate motor area (the anterior Rostral Cingulate Zone, RCZa) that is somatotopically organized. Whereas the face motor representations (mouth and eye) are located in the PCGS when present and in the CGS when the PCGS is absent (and, therefore, putatively in area 32′), the limb motor representations (hand and foot) are located in the CGS regardless of the presence or absence of the PCGS (and thus putatively in area 24c′)^31,32,37,50^. In exploratory situations in which the learning is driven by behavioral feedback, the analysis of visual, gustatory, and auditory feedback recruits a region located in the PCGS when present and in the CGS when the PCGS is absent^37,38,40^, a region that is co-localized with the face motor area of RCZa^37,50^. Altogether, these data led us to hypothesize that the role of the aMCC may be to perform an embodied analysis of feedback in exploratory situations, i.e. juice/visual/voice feedback recruit the face motor area of RCZa, whereas somatosensory feedback on the hand recruits the hand motor area of RCZa^37,50^. Within this framework, areas 32′ and 24c′ might support effector specific comparable feedback-related functional processes in exploratory situations. In rhesus macaques, recordings from the ventral and dorsal banks of the CGS have highlighted the role of both structures in reward processing and behavioral adaptation^54–56,58^. Given that both the cytoarchitectonic and the functional connectivity organization of the aMCC is comparable in macaque, chimpanzee and human brains, it is reasonable to hypothesize that the role of the aMCC in higher cognitive processing may also be preserved. However, in hemispheres displaying a PCGS, area 24c′ occupies about half of the dorsal bank of the CGS in human brains^10,44^, but it occupies only the fundus of the CGS and the most lateral part of the dorsal bank of the CGS in chimpanzee. One should be cautious in interpreting this difference as the boundaries between cingulate cortical areas 24c′ and 32′ in human brains are not consistent in the literature^59^.

Unlike the human brain^11,12,24,60^, we did not observe at the population level a left/right PCGS asymmetry in the chimpanzee. Although brain asymmetry is not a specifically human trait, its origins in a population have yet to be determined^61^. One hypothesis is that behavioral and brain asymmetry observed in primates might be related to the gradual evolution of language^62–64^. At the individual subject level, future studies should aim at investigating a putative link between PCGS morphology and cognitive abilities in chimpanzees, as has been done in humans^12,65^. Finally, another difference between human and chimpanzee brains is the prevalence of the PCGS in the ACC. While the PCGS is present in MCC in both chimpanzees and humans, the PCGS is more often observed in ACC in human brains. This finding suggests that differential evolutionary pressures impacted the ACC. This is a surprising result as some studies showed that the hotspot of cortical expansion in the primate medial frontal cortex may be located in the MCC rather than in the ACC^66,67^. Other studies, however, showed that the ACC presents high structural variability across subjects in several primate species, contrasting with the MCC which presents less variability^13,14,68^. The latter studies suggest that the ACC underwent greater expansion than the MCC during primate evolution. This expansion is however associated with a preserved cytoarchitectonic organization of the areas composing the ACC and adjacent ventromedial prefrontal cortex (vmPFC)^30,35,47^.

Within the ACC, resting-state analyses have shown that area 24c has stronger coupling with the anterior insula, the striatum and the ventrolateral prefrontal cortex, while perigenual area 32 has stronger coupling with the dorsolateral prefrontal cortex, the amygdala, and the hippocampus^33^. The ACC in the human brain has been associated with value-based computations in economic and social domains^69–74^. Similar properties have been identified in the non-human primate brain^52,75,76^. Implicit mentalizing abilities have been described in chimpanzees and macaques^77,78^, and are affected by reversible lesions of the ACC in macaques^78^. Recursive thinking and counterfactual manipulation of information to guide behavior have also been observed in macaques^75,79^. Counterfactual reasoning was also impacted by reversible lesions of the ACC in macaques^75^. However, human subjects have been shown to understand more complex relationships between social agents and their intentions than non-human primates^70,80^. The more complex the information about intentionality of social agents a human subject can comprehend, the larger the gray matter volume in the ACC and vmPFC^81^. Altogether, these results suggest that the building blocks of a human ACC have been present since the last common ancestor to human and macaques, but its evolution might reflect the development of recursive thinking in hominids^82^.

To conclude, using multimodal data, the present study demonstrates that chimpanzee brains do possess a PCGS in the MCC that is comparable to that in the human brain in terms of cytoarchitecture and functional connectivity. The similarities between ACC and MCC in primates and rodents are still a matter of debate^7,83^.

## Methods

### Subjects/specimens

#### Human subjects

The first step in this investigation was a reanalysis of the morphological organization of the PCGS in 197 human brains^13^ to refine our previous analysis by assessing the location and extent of the PCGS in the ACC and/or the MCC. High-resolution anatomical scans of these brains were obtained from the Human Connectome Project (HCP) database [http://www.humanconnectome.org/]. Only data from subjects with no family relationships were analyzed. The participants in the HCP study were recruited from the Missouri Family and Twin Registry that includes individuals born in Missouri^84^. Acquisition parameters of T1 anatomical scans are the following: whole head, 0.7 mm^3^ isotropic resolution, TR = 2.4 s, TE = 2.14 ms, flip angle = 8° (more details can be found at [https://humanconnectome.org/storage/app/media/documentation/s1200/HCP_S1200_Release_Appendix_I.pdf]). The full set of inclusion and exclusion criteria is detailed elsewhere. Briefly, the HCP subjects are healthy individuals free from major psychiatric or neurological illnesses. They are drawn from ongoing longitudinal studies^84^, in which they had received extensive assessments, including the history of drug use, and emotional and behavioral problems. The experiments were performed in accordance with relevant guidelines and regulations and all experimental protocols were approved by the Institutional Review Board (IRB) (IRB #201204036; Title: ‘Mapping the Human Connectome: Structure, Function, and Heritability’). All subjects provided written informed consent on forms approved by the Institutional Review Board of Washington University in St Louis. In addition, the present study received approval (n°15-213) from the Ethics Committee of Inserm (IORG0003254, FWA00005831) and from the Institutional Review Board (IRB00003888) of the French Institute of Medical Research and Health.

#### Chimpanzee (Pan troglodytes)

Three chimpanzee groups were examined in (1) the morphological sulcal organization of the medial frontal cortex (Group 1, *N* = 225), (2) in the cytoarchitectonic analysis of the MCC (Group 2, *N* = 3), and (3) in the functional connectivity analysis of the MCC (Group 3, *N* = 4, note that these four chimpanzees were also part of group 1).

Specifically, the morphological analysis aimed at refining our previous analysis on 225 chimpanzees^13^ by assessing the location and extent of the PCGS in the ACC and/or MCC. In the cytoarchitectonic analysis, we examined three post-mortem male chimpanzee brains that died from natural causes (CHIMP_1, CHIMP_2, and CHIMP_3; ages at death 38, 33, and 37 years, respectively). Within 14 h of each subject’s death, the brain was removed and immersed in 10% formalin at necropsy. In the in vivo resting-state fMRI study, we analyzed the data obtained in four chimpanzees: three females (CHIMP_A, CHIMP_B, CHIMP_C, respectively 16, 17, and 27 years of age at the time of the experiment) and one male (CHIMP_D, 15 years of age). All four chimpanzees were captive born and were members of the colony of apes housed at the Yerkes National Primate Research Center of Emory University.

The chimpanzees were all born in captivity and had all lived in social groups ranging from 2 to 13 individuals at the Yerkes National Primate Research Center and were housed according to institutional guidelines. Chimpanzee data collection was approved by the Institutional Animal Care and Use Committees at YNPRC and UTMDACC and followed the guidelines of the Institute of Medicine in the use of chimpanzees in research. We note here that all in vivo MRI scans were obtained prior to changes in NIH policy on the acquisition of neuroimaging data from chimpanzees (Nov 2015).

### MRI data acquisition

#### Structural MRI of post-mortem chimpanzee brains

Each post-mortem chimpanzee brain was scanned overnight in a 3 T Siemens Prisma MRI scanner to obtain structural T1 volumetric images (repetition time = 23 ms, echo time = 5.65 ms, voxel resolution = 0.4 × 0.4 × 0.4 mm). Each brain was scanned in a container filled with 10% formalin and supported with padding to prevent scanning artifacts from occurring near the edges of the container. 24, 4, and 36 repetitions of T1 scans of respectively CHIMP_1, CHIMP_2, and CHIMP_3 were obtained and averaged.

#### In vivo rs-fMRI acquisition in chimpanzee

In vivo resting-state functional magnetic resonance imaging (rs-fMRI) data came from Dr Hopkins’ laboratory. These data were acquired in early 2015 at the Yerkes National Primate Research Center (YNPRC) on the four adult chimpanzees at the time they were being surveyed for their annual physical examinations. Subjects were first immobilized by ketamine (10 mg/kg) or telazol (3–5 mg/kg) and subsequently anaesthetized with propofol (40–60 mg/[kg/h]) following standard procedures at the YNPRC. The subjects remained anaesthetized for the duration of the scans as well as the time needed to transport them between their home cage and the imaging facility (between 5 and 10 min). Chimpanzees were placed in the scanner chamber in a supine position with their head fitted inside the human-head coil.

They were scanned using a 3.0-T scanner (Siemens Trio; Siemens Medical Solutions USA, Inc., Malvern, PA, USA). T1-weighted images were collected using a three-dimensional gradient-echo sequence (repetition time = 2300 ms, echo time = 4.4 ms, number of signals averaged = 2, voxel resolution = 0.625 × 0.625 × 0.60 mm. In addition, 2 runs of 350 measurements each (16 min/session) of rs-fMRI scans were collected using a three-dimensional gradient-echo sequence (repetition time = 2683 ms, echo time = 25 ms, voxel resolution = 1.9 × 1.9 × 1.9 mm, right-left phase-encoding direction). An additional short run was performed with the same characteristics except that the phase-encoding was in the opposite direction (left-right). Scan duration was about 90 min.

After completing MRI procedures, the subjects were temporarily housed in a single enclosure for 6–12 h to allow the effects of the anesthesia to wear off before being returned to their social group.

### Sulcal morphology of the medial frontal cortex

On the basis of structural T1 MRI scans of each hemisphere of all chimpanzee and human brains, we established the presence or absence of a PCGS in each cerebral hemisphere. A PCGS was marked as present if running parallel and dorsal to the CGS^12,13^. We then examined whether this PCGS was located within the ACC, within the MCC, or extending along both regions. Note that the ACC-MCC limit was based on Vogt’s four-region model (Fig. 1)^15,45,85,86^.

#### Morphological analysis: occurrence and location of PCGS in human and chimpanzee

To establish the probability of occurrence of a PCGS in the MCC and the ACC in human and chimpanzee brains, we first reanalyzed the neuroimaging T1 anatomical data of 197 human brains and 225 chimpanzee brains from our previous study^13^. From this inspection, we identified 76 chimpanzees displaying a PCGS at least in one hemisphere (15, 29, and 32 displaying a PCGS in both hemispheres, only in the left hemisphere, and only in the right hemispheres, respectively), for a total of 91 hemispheres displaying a PCGS. We also identified 139 human brains displaying a PCGS at least in one hemisphere (45, 69, and 25 displaying a PCGS in both hemispheres, only in the left hemisphere, and only in the right hemispheres, respectively), for a total of 184 hemispheres displaying a PCGS. In these hemispheres, we then identified whether the PCGS was present in the ACC, the MCC, or in both the ACC and MCC. The limit between the ACC and the MCC was identified using the probabilistic cytoarchitectonic map of the ACC from the JuBrain atlas (https://jubrain.fz-juelich.de/apps/cytoviewer2/cytoviewer-main.php#)^33^.

#### Cytoarchitectonic analysis: occurrence and location of PCGS in chimpanzee

From the morphological inspection of the three chimpanzee brains included in this analysis, we selected four hemispheres for the cytoarchitectonic analysis:

The left hemisphere of CHIMP_1, in which the PCGS is absent.

The right hemisphere of CHIMP_1, which displays a PCGS.

The left hemisphere of CHIMP_2, where a PCGS is present in the anterior-most part of the aMCC, but absent in the posterior part of the aMCC.

The right hemisphere of CHIMP_3, in which the PCGS is absent.

Note that the remaining hemispheres (left hemisphere of CHIMP_3 and right hemisphere of CHIMP_2) displayed no PCGS.

#### Rs-fMRI analysis: occurrence and location of PCGS in chimpanzee

We identified three hemispheres with a PCGS in the four chimpanzees included in this analysis:

Both the left and right hemispheres of CHIMP_C, and the left hemisphere of CHIMP_A, displayed a PCGS.

The right hemisphere of CHIMP_A, and both hemispheres of CHIMP_D and CHIMP_B did not display a PCGS.

### Blocking and histological processing

Based on the averaged structural MRI data of the chimpanzee brains and using a neuronavigation system (Brainsight), each selected brain was cut in several blocks to obtain histological sections optimal for the study of the architecture of the regions of interest (see Novek et al.^87^ for the method used). The blocks including the MCC were optimized to allow for histological sections that were perpendicular to the orientation of both the cingulate and/or paracingulate sulci. Note that the remaining blocks were processed for ongoing studies aiming to assess cytoarchitectonic areas of various regions of the chimpanzee cortex. All blocks were cryoprotected by immersion in buffered sucrose solutions from 10 to 30% until they sank, frozen to −60 ˚C in a bath of 2-methylbutane chilled by a surrounding mixture of dry ice and ETOH, then stored at −80 ˚C until use, where the block was kept surrounded by dry ice on a frozen microtome stage during sectioning. The histological sections were cut at a thickness of 30 μm, three out of every ten sections were kept, and a photograph was taken before each set of the kept sections throughout the entire blocks to aid with 3D reconstructions.

### Cytoarchitectonic analysis

The detailed cytoarchitectonic analysis of the mid-cingulate cortex of the selected blocks was carried out from the sections that were cut in a coronal orientation. Every tenth section was mounted on 2” × 3” coated slides and stained with cresyl violet, a Nissl cell body stain, for cytoarchitectonic analysis; the remaining sections are being used in ongoing studies.

The architectonic organization of the MCC and, more specifically, the boundaries between the cytoarchitectonic areas composing the MCC, were identified on high-resolution tilted images of the entire region of interest from the cresyl violet sections, obtained under bright field with a Zeiss Axio Scan.Z1 at ×10 magnification.

The assessment of the architectonic organization of the MCC was based on prior studies of this region in both the human^10^ and in the macaque monkey^30,88^ brains. Proceeding from the corpus callosum in a dorsal direction towards the lateral surface of the frontal lobe, the following areas were identified in the cingulate region: area 24a′, area 24b′, area 24c′, and area 32′. Note that, dorsal to these cingulate cortical areas, the medial frontal areas 6. 8, and 9 are located. Area 24a′ is an agranular area located on the gyrus just dorsal to the corpus callosum. It is characterized by a clear layer II, a thick layer III, a dense layer V, and a poorly defined layer VI. Area 24b’ is an agranular area located dorsal to area 24a′ and ends where the CGS starts. It is characterized by a broad layer III containing distinct layers IIIa, b, and c, as well as a highly prominent layer V composed of large pyramidal neurons. Area 24c′ is an agranular area located in the ventral bank of the CGS and is characterized by thin layers II and III, as well as the presence of more densely packed large pyramidal neurons in layer V in comparison with area 24b’. Finally, area 32′ is a dysgranular cingulo-frontal transitional area located dorsal to area 24c′ in the dorsal bank of the CGS when there is no PCGS in the human brain and located within the PCGS if present^10^. In the macaque, area 32′ (labeled as 32/6 or 32/8 in macaque) is located in the dorsal bank of the CGS ^30,88^. It displays a wide layer IIIc containing large pyramidal neurons, a dysgranular layer IV, and a thinner and less dense layer V than in area 24c′.

### Rs-fMRI data analysis

Data were collected using 2 phase-encoding directions (2 full runs in Right-Left, and a shorter 3d run in Left-Right directions). It resulted in two pairs of images with distortions going in opposite directions (pair 1: 1^st^ run in right-left and 3d run in left-right direction; pair 2: 2^d^ run in right-left and 3d run in left-right direction). Distortions were corrected using TOPUP’s FSL tool. First, TOPUP estimated from these pairs the susceptibility-induced off-resonance field using a method similar to that described by Andersson et al.^89^ as implemented in FSL^90^. Once estimated, the images were then combined into corrected ones. Two runs, corrected for distortions, resulted from this analysis and were further preprocessed.

The preprocessing of resting-state scans was then performed with SPM 12. The first 5 volumes of each run were removed to allow for T1 equilibrium effects. The head motion correction was applied using rigid body realignment and we then applied a slice timing correction using the time center of the volume as reference. Then, using the AFNI software^91^, the segmentation of each brain was performed on skull-stripped brains. A temporal filtering was then applied to extract the spontaneous slowly fluctuating brain activity (0.01–0.1 Hz). Finally, linear regression was used to remove nuisance variables (the cerebrospinal fluid and white matter signals from the segmentation) and spatial smoothing with a 4-mm FWHM Gaussian kernel was applied to the output of the regression.

#### Seed selection in the MCC

The seeds consisted of 2.5-mm radius spheres and were positioned in the aMCC at the same antero-posterior level as in the cytoarchitectonic study as follows:

In hemispheres displaying no PCGS (*N* = 5): a seed assigned to area 24c′ was positioned in the ventral bank of the CGS and a seed assigned to area 32′ was positioned in the dorsal bank of the CGS.

In hemispheres displaying a PCGS (*N* = 3): a seed assigned to area 24c′ was positioned in the CGS and a seed for area 32′ was positioned in the ventral bank of the PCGS.

In both cases, the two seeds were positioned on an imaginary line going through the posterior limit of the genu of the corpus callosum and perpendicular to the axis on which the CGS and PCGS are running (see Supplementary Fig. 1 for positioning of seeds in all chimpanzees and hemispheres). In hemispheres displaying no PCGS, the two seeds (area 24c′ and area 32′) displayed a small overlap. This overlap was removed before performing the ROI-based resting-state data analysis.

#### Selection of regions of interest (ROIs)

For a stricter comparison of the present results with results obtained in our previous study which assessed the functional connectivity of the CGS and PCGS in the anterior part of the human MCC^31^, we used the same ROIs (see below and Supplementary Fig. 1 for positioning of ROIs in all chimpanzees and hemispheres). Each ROI consisted of a sphere with a 5-mm radius.

### ROIs selection in motor cortical areas

For each subject, 3 ROIs within the motor cortex of both hemispheres were identified based on sulcal morphology. These included the hand motor region (the precentral knob) within the central sulcus –M1Hand–^43^ and the primary face motor region within the ventral part of the posterior part of the precentral gyrus –M1Face–^92^. We also included the frontal eye field –FEF–. In the human brain, this region is located within the ventral branch of the superior precentral sulcus^39^. As the chimpanzee presents the same sulcal pattern in this region, including a ventral branch of the superior precentral sulcus, we tentatively included this ROI in our analysis.

### Selection of ROIs in the prefrontal cortex

For each subject, 6 ROI locations within the left prefrontal cortex were identified based on the local anatomy. On a rostro-caudal axis:

The frontopolar cortex –Area 10–. In the human brain, this region is located at the intersection between the vertical segment of the intermediate frontal sulcus, the lateral and the medial frontomarginal sulcus, see^41^. Because chimpanzee brains display a similar sulcal pattern, we hypothesized that Area 10 most likely lies at the same location.

The dorsolateral prefrontal cortex -DLPFC-. In the human brain, area 9/46 of the DLPFC lies at the rostral level of the genu of the corpus callosum, above the inferior frontal sulcus. Because the chimpanzee displays also an inferior frontal sulcus that is also present at the level of the rostral level of the genu of the corpus callosum, we hypothesized that area 9/46 lies at the same location.

Broca’s region: The two cytoarchitectonic areas that comprise Broca’s region, namely area 44 and area 45, have been shown to be located in the chimpanzee brain between the inferior precentral sulcus and the fronto-orbital sulcus, and anterior to the fronto-orbital sulcus^42,93^.

The frontal operculum –Fo– (intersection between the frontal operculum and the circular sulcus, see ref. ^38^.

#### Statistics and reproducibility

The mean signal from Seeds and ROI regions was then extracted using AFNI software. For each chimpanzee brain, correlation coefficients between the two seeds with the various ROIs in the prefrontal cortex and the motor cortex were computed and normalized using the Fisher’s r-to-z transform formula. The significant threshold at the individual subject level was *Z* = 0.1 (*p* < 0.05). These normalized correlation coefficients, which corresponded to the functional connectivity strength between each seed and each ROI in individual chimpanzee brains, were subsequently processed with R statistical software (https://www.r-project.org/) for all the following analyses.

To compare the connectivity profile of each seed with the various lateral frontal ROIs, we constructed boxplots corresponding to the correlation strength of each seed location with each of the ROIs. Based on these boxplots, it can be discerned that both Area 24c′ and Area 32′ seeds have stronger connectivity with prefrontal regions and weaker connectivity with premotor and motor areas (see “Results”). We then characterized this rostro-caudal functional axis based on the correlation profiles of Area 24c′ and Area 32′, when a PCGS is present and also when it is not present, with the lateral frontal cortex by estimating linear trends in the correlation strength for each seed with the rostro-caudal lateral frontal ROIs (for details, see “Methods“ in Loh et al.^31^). The 8 ROIs were first ranked along a rostro-caudal axis based on their average Y coordinate values across chimpanzee brains and recoded into a numeric axis variable (ROIline): Area 10 (most anterior)-1, DLPFC-2, Area 45-3, Area 44-4, Fo-5, FEF-6, M1Face-7, M1Hand (most posterior)-8. We then performed multiple linear regressions on the correlation *z* values with seed identity (area 24c′ and area 32′), sulcal morphology (PCGS absent or present), and the linear axis variable (ROIline) as predictors. We assessed whether the linear trends (slopes) observed for each seed were identical or not in each sulcal morphology using a 3-ways ANOVA.

### Reporting summary

Further information on research design is available in the Nature Research Reporting Summary linked to this article.

## Supplementary information

Supplementary Information

Description of Additional Supplementary Files

Supplemental Data 1

Supplemental Data 2

Reporting Summary

## Data Availability

The source data underlying Figs. 1 and 5 are provided as Source Data file (respectively Supplementary Data 1 and Supplementary Data 2). Human anatomical MRI scans are available from the Human Connectome Project database [http://www.humanconnectome.org/]^94^, and Chimpanzee anatomical MRI and rs-fMRI scans are available from Dr. W. Hopkins [http://www.chimpanzeebrain.org/]^95^. A reporting summary for this Article is available as a Supplementary Information file.
