# Supplementary material for: Chimpanzee histology and functional brain imaging show that the paracingulate sulcus is not human-specific
Source: Commun Biol. 2021 Jan 8;4:54. doi: 10.1038/s42003-020-01571-3 (PMC7794552; doi:10.1038/s42003-020-01571-3)
Supplement: Supplementary file 1 — Supplementary Information [file 42003_2020_1571_MOESM1_ESM.pdf]

a. Hemispheres displaying a PCGS

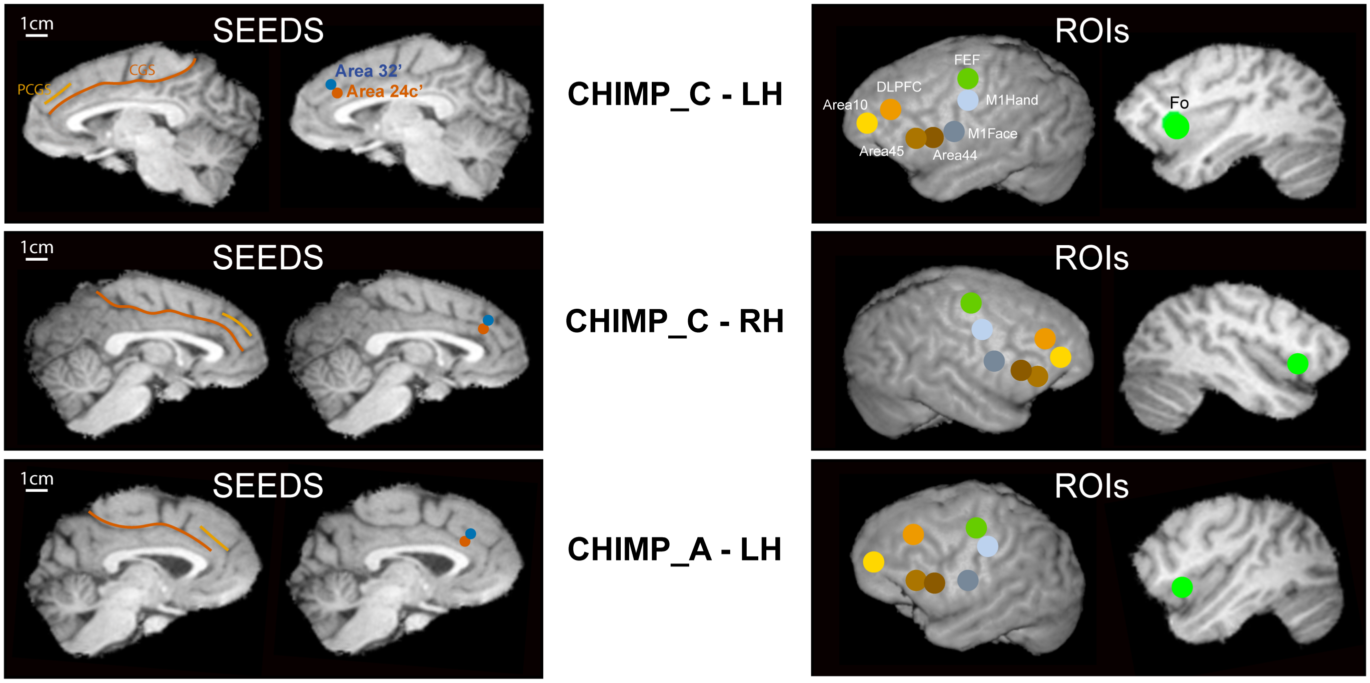

b. Hemispheres displaying no PCGS

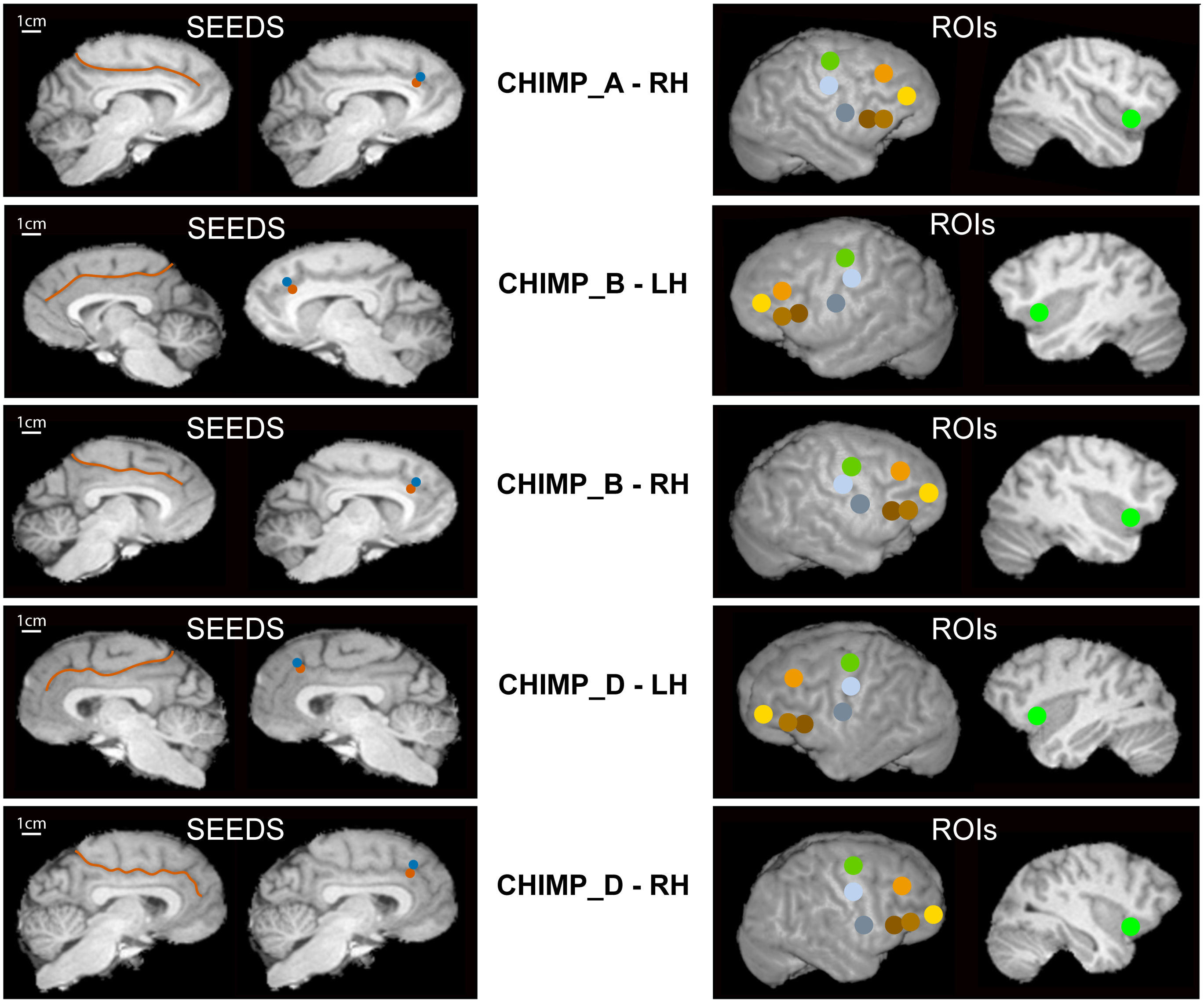

Supplemental Figure 1. Seeds and ROIs used for the rs-fMRI data analysis in hemispheres displaying a PCGS (a) or not (b). Abbreviations: LH, left hemisphere; RH, right hemisphere; DLPFC, dorsolateral prefrontal cortex; Fo, frontal operculum; FEF, frontal eye field.
