# Supplementary material for: Chimpanzee histology and functional brain imaging show that the paracingulate sulcus is not human-specific
Source: Commun Biol. 2021 Jan 8;4:54. doi: 10.1038/s42003-020-01571-3 (PMC7794552; doi:10.1038/s42003-020-01571-3)
Supplement: Supplementary file 2 — Description of Additional Supplementary Files [file 42003_2020_1571_MOESM2_ESM.pdf]

Description of Additional Supplementary Files

File Name: Supplementary Data 1
Description: **Source data underlying Fig 1.** In columns from 1 to 4 are indicated, respectively, the specie (human or chimpanzee), the subject ID, the brain hemisphere, and the extent of the paracingulate sulcus (PCGS, in ACC, MCC or in both ACC/MCC).

File Name: Supplementary Data 2
Description: **Source data underlying Fig 5.** In columns from 1 to 12 are indicated, respectively, the chimpanzee ID, the brain hemisphere, the seed ID, the ROI ID, the run (1 or 2), the voxel ID, the voxel X coordinates, the voxel Y coordinate, the voxel Z coordinate, the correlation Z value between the seed and the voxel of the ROI assessed, the morphology (PCGS present or absent) and the ROIline (values from 1 to 8).
