# Supplementary material for: Chimpanzee histology and functional brain imaging show that the paracingulate sulcus is not human-specific
Source: Commun Biol. 2021 Jan 8;4:54. doi: 10.1038/s42003-020-01571-3 (PMC7794552; doi:10.1038/s42003-020-01571-3)
Supplement: Supplementary file 5 — Reporting Summary [file 42003_2020_1571_MOESM5_ESM.pdf]

nature research

Corresponding author(s): AMIEZ CELINE

Last updated by author(s): Nov 13, 2020

Reporting Summary

Nature Research wishes to improve the reproducibility of the work that we publish. This form provides structure for consistency and transparency in reporting. For further information on Nature Research policies, see our Editorial Policies and the Editorial Policy Checklist.

Statistics

For all statistical analyses, confirm that the following items are present in the figure legend, table legend, main text, or Methods section.

n/a	Confirmed	
☐	☒	The exact sample size (*n*) for each experimental group/condition, given as a discrete number and unit of measurement
☐	☒	A statement on whether measurements were taken from distinct samples or whether the same sample was measured repeatedly
☐	☒	The statistical test(s) used AND whether they are one- or two-sided<br>*Only common tests should be described solely by name; describe more complex techniques in the Methods section.*
☒	☐	A description of all covariates tested
☐	☒	A description of any assumptions or corrections, such as tests of normality and adjustment for multiple comparisons
☐	☒	A full description of the statistical parameters including central tendency (e.g. means) or other basic estimates (e.g. regression coefficient) AND variation (e.g. standard deviation) or associated estimates of uncertainty (e.g. confidence intervals)
☐	☒	For null hypothesis testing, the test statistic (e.g. *F*, *t*, *r*) with confidence intervals, effect sizes, degrees of freedom and *P* value noted<br>*Give P values as exact values whenever suitable.*
☒	☐	For Bayesian analysis, information on the choice of priors and Markov chain Monte Carlo settings
☒	☐	For hierarchical and complex designs, identification of the appropriate level for tests and full reporting of outcomes
☒	☐	Estimates of effect sizes (e.g. Cohen's *d*, Pearson's *r*), indicating how they were calculated

Our web collection on statistics for biologists contains articles on many of the points above.

Software and code

Policy information about availability of computer code

Data collection	NA
Data analysis	Normalization of human and chimpanzee brains was performed with SPM12 (https://www.fil.ion.ucl.ac.uk/spm/software/spm12/). rs-fMRI data analysis was performed with AFNI software (https://afni.nimh.nih.gov).<br>All statistics were performed with R software, R Development Core Team under R-Studio.

For manuscripts utilizing custom algorithms or software that are central to the research but not yet described in published literature, software must be made available to editors and reviewers. We strongly encourage code deposition in a community repository (e.g. GitHub). See the Nature Research guidelines for submitting code & software for further information.

Data

Policy information about availability of data

All manuscripts must include a data availability statement. This statement should provide the following information, where applicable:

- Accession codes, unique identifiers, or web links for publicly available datasets
- A list of figures that have associated raw data
- A description of any restrictions on data availability

Anatomical (T1) neuroimaging data of human brains are available from the Human Connectome Project (humanconnectome.org) database. Chimpanzee MRI data are available upon request to William Hopkins (http://www.chimpanzeebrain.org/).

April 2020

Field-specific reporting

Please select the one below that is the best fit for your research. If you are not sure, read the appropriate sections before making your selection.

☒ Life sciences ☐ Behavioural & social sciences ☐ Ecological, evolutionary & environmental sciences

For a reference copy of the document with all sections, see nature.com/documents/nr-reporting-summary-flat.pdf

Life sciences study design

All studies must disclose on these points even when the disclosure is negative.

Sample size	Cytoarchitectonic analysis: 3 chimpanzee brains.<br>Resting-state fMRI analysis: 4 chimpanzee brains.<br>Morphological analysis: 225 chimpanzee brains and 197 human brains.
Data exclusions	No exclusion.
Replication	NA
Randomization	Randomization is not pertinent in our study. Human brains and chimpanzee brains are treated separately.
Blinding	Blinding across group is also not relevant, primate brains being analysed separately.

Reporting for specific materials, systems and methods

We require information from authors about some types of materials, experimental systems and methods used in many studies. Here, indicate whether each material, system or method listed is relevant to your study. If you are not sure if a list item applies to your research, read the appropriate section before selecting a response.

Materials & experimental systems

n/a	Involved in the study	
☒	☐	Antibodies
☒	☐	Eukaryotic cell lines
☒	☐	Palaeontology and archaeology
☐	☒	Animals and other organisms
☐	☒	Human research participants
☒	☐	Clinical data
☒	☐	Dual use research of concern

Methods

n/a	Involved in the study	
☒	☐	ChIP-seq
☒	☐	Flow cytometry
☐	☒	MRI-based neuroimaging

Animals and other organisms

Policy information about studies involving animals; ARRIVE guidelines recommended for reporting animal research

Laboratory animals	Chimpanzee (pan troglodytes)
Wild animals	None
Field-collected samples	NA
Ethics oversight	We did not aquire novel in-vivo data in the present article. Rather, data correspond to existing databases in the laboratories of the authors involved. Note however that data collected initially for studies on Chimpanzees were approved by the Institutional Animal Care and Use Committees at YNPRC and UTMDACC and also followed the guidelines of the Institute of Medicine on the use of chimpanzees in research.

Note that full information on the approval of the study protocol must also be provided in the manuscript.

Human research participants

Policy information about studies involving human research participants

Population characteristics	Healthy human subjects from the Human Connectome Database.
Recruitment	The full set of inclusion and exclusion criteria is detailed in Van Essen et al. (2012). In short, the HCP subjects are healthy

Recruitment	individuals who are free from major psychiatric or neurological illnesses. They are drawn from ongoing longitudinal studies, where they received extensive previous assessments including the history of drug use, emotional, and behavioral problems.
Ethics oversight	The experiments mentioned in the Human Connectome Database were performed in accordance with relevant guidelines and regulations and all experimental protocol was approved by the Institutional Review Board (IRB) (IRB # 201204036; Title: 'Mapping the Human Connectome: Structure, Function, and Heritability'). Furthermore, this project received approval (n° 15-213) from the ethic committee of Inserm (IORG0003254, FWA00005831) and from the Institutional Review Board (IRB00003888) of the French institute of medical research and health.

Note that full information on the approval of the study protocol must also be provided in the manuscript.

Magnetic resonance imaging

Experimental design

Design type	None
Design specifications	None
Behavioral performance measures	None

Acquisition

Imaging type(s)	In-vivo MRI scans: Structural data came from existing databases.<br>Post-mortem MRI scans: structural data aquired at the Montreal Neurological Institute.
Field strength	3T and 1.5T
Sequence & imaging parameters	Human: only structural sequences were used.<br>Chimpanzee: in-vivo MRI scans included both structural and resting-state fMRI sequences. Post-mortem MRI scans included only structural MRI sequences.
Area of acquisition	Whole brain imaging

Diffusion MRI ☐ Used ☒ Not used

Preprocessing

Preprocessing software	Structural data in both human and chimpanzee: spm12<br>rs-fMRI data in chimpanzee: FSL, spm12, and AFNI
Normalization	All primate in-vivo structural data were normalized.
Normalization template	Human brains were normalized in the human MNI steretotaxic coordinate system (http://www.bic.mni.mcgill.ca/ServicesAtlases/HomePage) . Chimpanzee brains were normalized in the chimpanzee standard brain developed by Dr. W. Hopkins (Hopkins and Avants 2013, available at www.chimpanzeebrain.org).
Noise and artifact removal	Chimpanzee rs-fMRI data were collected using 2 phase-encoding directions (2 full runs in Right-Left, and a shorter 3d run in Left-Right directions). It resulted in two pairs of images with distortions going in opposite directions (pair 1: 1st run in right-left and 3d run in left-right direction; pair 2: 2d run in right-left and 3d run in left-right direction). Distortions were corrected using TOPUP's FSL tool.
Volume censoring	None

Statistical modeling & inference

Model type and settings	NA
Effect(s) tested	NA

Specify type of analysis: ☒ Whole brain ☐ ROI-based ☐ Both

Statistic type for inference (See Eklund et al. 2016)	NA
Correction	NA

Models & analysis

n/a	Involved in the study
☐	☒ Functional and/or effective connectivity
☒	☐ Graph analysis
☒	☐ Multivariate modeling or predictive analysis

Functional and/or effective connectivity

For each chimpanzee brain involved in the rs-fMRI study, correlation coefficients between the two seeds (area 24c' and area 32') with the various ROIs in the lateral prefrontal cortex and the motor cortex were computed and normalized using the Fisher's r-to-z transform formula. The significant threshold at the individual subject level was Z = 0.1 ($p < 0.05$). These normalized correlation coefficients, which corresponded to the functional connectivity strength between each seed and each ROI in individual brains, were subsequently processed with R statistical software.
